# Local anesthetic levobupivacaine promotes hip-fracture healing by targeting HIF/Ca^2+^signaling

**DOI:** 10.1016/j.clinsp.2025.100831

**Published:** 2025-11-11

**Authors:** Hui Zhang, Ying Zhang, Fang Wang, Yulong Wei, Dezhi Wang

**Affiliations:** Department of Anesthesiology, Honghui Hospital, Xi’an Jiaotong University, Xi’an, China

**Keywords:** Levobupivacaine, Bone fracture healing, Mesenchymal stem cells, HIF-1α

## Abstract

**Background:**

Bone fractures, characterized by structural discontinuities in bone tissue, are a leading cause of functional impairment and reduced quality of life. Levobupivacaine, a long-acting local anesthetic, is routinely employed in perioperative settings for fracture management, including surgical anesthesia and postoperative analgesia. However, its potential influence on bone fracture healing remains underexplored. This study aims to evaluate the effects of levobupivacaine on the molecular and cellular mechanisms underlying bone fracture repair.

**Methods:**

A rat model with a bone fracture was established and treated with levobupivacaine. The images of the bone site were scanned by micro-CT and bone parameters were calculated. The tissue structure and healing were detected by Hematoxylin/Eosin (HE) staining. Expression of VEGF, CD90, and HIF-1α in bone tissues was measured by Immunohistochemistry (IHC) analysis. Bone Marrow-derived Mesenchymal Stem Cells (BMSCs) were isolated from the bones of rats. Cell migration was assessed using by Transwell system. The content of calcium in rat blood samples and cells was detected. The protein level of HIF-1α was detected by western blotting*.*

**Results:**

Levobupivacaine facilitated the bone fracture healing, manifested by increased formation of callus and elevated Total Volume (TV), Bone Volume (BV), Bone Mineral Density (BMD) to BMD/BV, TV ratio, and BV/TV. Further, Levobupivacaine promoted the CD90, HIF-1α, and VEGF expression in bone tissues during healing. The proliferation, migration, and calcium production were elevated by levobupivacaine treatment, and HIF-1α depletion suppressed these effects.

**Conclusion:**

Levobupivacaine facilitates bone healing by promoting migration and proliferation of BMSCs through activating HIF-1α/Ca^2+^ signaling. These findings identify the clinical potential of levobupivacaine in the treatment of bone fractures.

## Highlights

• Levobupivacaine is a common local anesthetic agent for pain management.
• Bone fracture rat model was established and treated with levobupivacaine.
• Levobupivacaine for bone fracture healing, promoted CD90, HIF-1α, VEGF expression.
• Proliferation, migration, and calcium production were elevated by levobupivacaine.
• Proliferated bone marrow-derived mesenchymal stem cells by HIF-1α/Ca^2+^ signaling.

## Introduction

Hip fractures pose a significant threat to the health and survival of elderly populations. Owing to age-related anatomical and physiological changes, fracture healing in this region is frequently protracted.^1^ A substantial proportion of patients exhibit delayed union, which can lead to persistent functional deficits and a marked decline in quality of life.^2^ The fracture healing process consists of three distinct phases: 1) Hematoma formation and inflammation, 2) Primary callus formation, and 3) Callus remodeling. These stages involve localized bleeding, inflammation, mesenchymal cell differentiation, and extracellular matrix deposition, culminating in the restoration of bone structure and function. Despite surgical intervention and conventional therapies, 5 %–10 % of fractures result in delayed union or nonunion.^3^

In recent decades, Mesenchymal Stem Cells (MSCs) have gained significant attention as a promising therapeutic strategy in regenerative medicine, particularly for augmenting fracture healing. These cells demonstrate multipotent differentiation potential and serve as critical regulators of bone metabolism and homeostasis.^4^ Beyond their osteogenic potential, MSCs contribute to fracture healing through paracrine signaling, secreting bioactive molecules that promote angiogenesis, modulate inflammation, and facilitate extracellular matrix remodeling.^5^ These cells express osteogenic markers such as Alkaline Phosphatase (ALP), Osteocalcin (OCN), and type I Collagen (COL1), indicating their differentiation into mature osteoblasts.

MSCs orchestrate bone healing through their secretory profile, releasing key regulatory molecules such as Transforming Growth Factor-Beta (TGF-β), Vascular Endothelial Growth Factor (VEGF), Fibroblast Growth Factor (FGF), and Platelet-Derived Growth Factor (PDGF).^6^, ^7^, ^8^, ^9^, ^10^, ^11^, ^12^ These factors enhance angiogenesis, cell recruitment, proliferation, and differentiation, collectively fostering a conducive microenvironment for bone regeneration. Additionally, MSCs exhibit immunomodulatory properties, mitigating excessive inflammation and immune rejection.^13^^,^^14^ Their secretion of anti-inflammatory mediators such as Interleukin-10 (IL-10) helps modulate the inflammatory milieu, optimizing conditions for tissue repair. Furthermore, MSCs upregulate Intercellular Adhesion Molecule-1 (ICAM-1) and Vascular Cell Adhesion Molecule-1 (VCAM-1), inhibiting T-cell activation and leukocyte recruitment to injury sites.^15^ These paracrine interactions underscore the indirect yet critical role of MSCs in bone healing.^16^

Hypoxia-Inducible Factor-1 (HIF-1), a transcription factor composed of HIF-1α and HIF-1β subunits, plays a central role in cellular responses to hypoxia. Under normoxic conditions, HIF-1α undergoes hydroxylation by Prolyl Hydroxylases (PHDs), leading to its proteasomal degradation. In hypoxia, PHD activity is suppressed, allowing HIF-1α stabilization, nuclear translocation, and dimerization with HIF-1β. This complex regulates downstream genes involved in angiogenesis, cell survival, and metabolism.^17^ Emerging evidence suggests that HIF-1α is a key regulator of MSC migration and osteogenic differentiation during fracture healing.^18^

Local anesthetics, such as levobupivacaine, are primarily used for perioperative pain management in orthopedic procedures. However, recent studies indicate that levobupivacaine may influence bone regeneration and recovery beyond analgesia.^19^ While its effects on wound healing appear biphasic ‒ initially inhibitory but later promotive ‒ levobupivacaine has been proposed as a suitable anesthetic for late-stage wound repair.^20^ Moreover, sustained-release formulations of levobupivacaine, delivered via electrosprayed microparticles, provide prolonged analgesia at fracture sites for over 12-days, suggesting potential therapeutic benefits.^21^ Nevertheless, the precise mechanisms underlying its impact on fracture healing remain under investigation.

This study aims to elucidate the therapeutic potential of levobupivacaine in fracture healing. The authors hypothesize that levobupivacaine enhances bone repair in vivo by promoting MSC proliferation and migration while activating the HIF-1α/Ca^2+^ signaling pathway.

## Materials and methods

### Model of a rat bone fracture

Eight-week-old male SD rats weighing about 200 g were bought from Vital River Laboratory (Beijing, China) and used in the experiment. All animal experiments were approved by the Ethics Committee of the Honghui Hospital, Xi’an Jiaotong University (Approval n° MDL2021–10–10–01) and performed in accordance with the Guidelines for the Care and Use of Laboratory Animals. The rats were randomly allocated into three groups (n = 6/group): control, model, and model + levobupivacaine treatment. Following intraperitoneal anesthesia with 3 % sodium pentobarbital (50 mg/kg), rats were positioned supine on a surgical platform with limbs secured. The operative site was shaved, disinfected with povidone-iodine, and draped with sterile surgical towels to maintain aseptic conditions while exposing the surgical field. Make a 1.5–2 cm longitudinal incision on the lateral femur. Bluntly dissect the mucosa and muscles to expose the midshaft of the femur. Rinse the surgical site with saline. Transect the femur at the mid-diaphysis using an electric bone saw, then perform intramedullary fixation with a 1.2 mm Kirschner wire. Secure the fracture ends with traction fixation using 3‒0 absorbable sutures. Irrigate the wound thoroughly with 0.9 % sodium chloride solution, followed by layered closure of the muscle and skin. Postoperatively, administer penicillin via intramuscular injection at 100,000 units per animal. Continue injections for 3 consecutive days to prevent infection. Levobupivacaine (50 µmoL/kg body weight) was administered through the tail vein once a day for 20 days.^22^

### Micro-CT analysis

After the wire was carefully removed, the structure of the femur was scanned using a micro-CT system (Scanco Medical, Switzerland), and bone parameters were analyzed using Micro-CT software. For quantitative analysis, cross-sectional images of the osteotylus were selected, and bone parameters were calculated using the integrated Micro-CT analysis software. The evaluated parameters included: Bone Volume (BV), Total Volume (TV), and Bone Mineral Density (BMD) to TV, BMD to BV, and BV to TV. All measurements were performed in triplicate to ensure reproducibility, and the results were averaged for statistical analysis.

### Histological analyses

The femurs underwent isolation, fixation in 4 % PFA, decalcification, and paraffin embedding to create serial sections with a thickness of 5 μm. The tissue damage and structure were observed by Hematoxylin/Eosin (HE) staining (Beyotime, China) in line with the manufacturer’s description. The expression of CD90, HIF-1α, and VEGF in bone tissues was measured by Immunohistochemistry (IHC) analysis. In brief, the tissue slices were dewaxed, blocked with goat serum, and placed overnight at 4 °C in a solution containing primary antibodies. The next day, the tissues were visualized after incubation with HRP-labeled anti-rabbit secondary antibody (Abcam, USA) and reaction with 3,3′-diaminobenzidine (DAB, Beyotime, China). Hematoxylin was used as a counterstain for the nuclei. The images were observed by a microscope (Leica, Germany).

### Isolation and culture of BMSCs

From the bones of SD rats, the BMSCs were isolated. The SD rats were euthanized by cervical dislocation and immediately immersed in 75 % ethanol for surface disinfection. After removal, the limbs were wiped sequentially with iodine tincture and alcohol-soaked cotton balls. Using sterile surgical instruments, the femurs and tibias were carefully dissected, with all attached soft tissues removed, followed by extensive PBS washing. The bones were then placed in DMEM/F-12 medium. Both epiphyses were excised, and the marrow cavity was flushed repeatedly with DMEM medium using a n° 5-gauge needle inserted at one end while collecting effluent from the opposite extremity. The bone marrow wash was mechanically dissociated by repeated pipetting to obtain a single-cell suspension, which was centrifuged at 1000 × *g* for 5 min at room temperature. After supernatant removal, the cell pellet was resuspended in DMEM/F-12 medium supplemented with 15 % FBS. The isolated BMSCs were placed in a constant temperature incubator at 37 °C with 5 % CO_2_ for cultivation and used within 8 passages. The siRNA targeting HIF-1α was synthesized by Ribo Bio (China) and transfected into BMSCs using Lipofectamine 2000.

### CCK-8 assay

overnight to promote attachment. Following that, levobupivacaine (5 µM) was applied to the cells for a full day. After adding 10 μL of CCK-8 reagent (Beyotime, China) to each well, the plate was incubated at 37 °C with 5 % CO_2_ for 2 h. Absorbance was measured at 450 nm using a microplate reader, and cell viability was calculated based on the recorded values.

### Colony formation assay

One thousand BMSCs were sown into each well of six-well plates after being suspended in full media as single cells. The cells were cultured for two weeks before any discernible colonies formed. After that, the cells were fixed with 2 mL methanol per well for 30 min at room temperature. After methanol removal, cells were stained with 2 mL 0.1 % crystal violet solution for 3 min, followed by gentle washing with PBS. Colonies were photographed using a digital imaging system and quantified by manual counting under an optical microscope.

### Cell migration

Cell migration was measured by the Transwell system (Corning, USA). BMSCs were placed in the upper chambers of Transwell in serum-free medium, and the lower chamber was filled with normal culture medium. After incubation for 48 h, the chambers were collected, and the cells inside the membranes were wiped out. Cells that migrated through membranes were stained with 0.5 % crystal violet and captured under a microscope (Leica, Germany).

### Detection of Ca^2+^

Blood samples were collected from the tail vein of rats. After centrifuging, the supernatant was subjected to Calcium Colorimetric Assay Kit (Beyotime, China) for detection of Ca^2+^ in accordance with the manufacturer’s protocol. For measurement of intracellular Ca^2+^, BMSCs were incubated with Fluo-3 AM probe at 37 °C for 30 min. The fluorescence was monitored by using a microplate detector (Thermo, USA).

### Statistics

The information was presented as the mean ± Standard Deviation (SD) of three separate replicates. Software called SPSS 19.0 was used to perform statistical analysis. Tukey's post-hoc test was used to assess the differences between two or more groups using the Student's *t*-test or one-way ANOVA test; p < 0.05 was used to indicate statistical significance.

## Results

### Levobupivacaine improves bone fracture healing

**Fig. 1:**
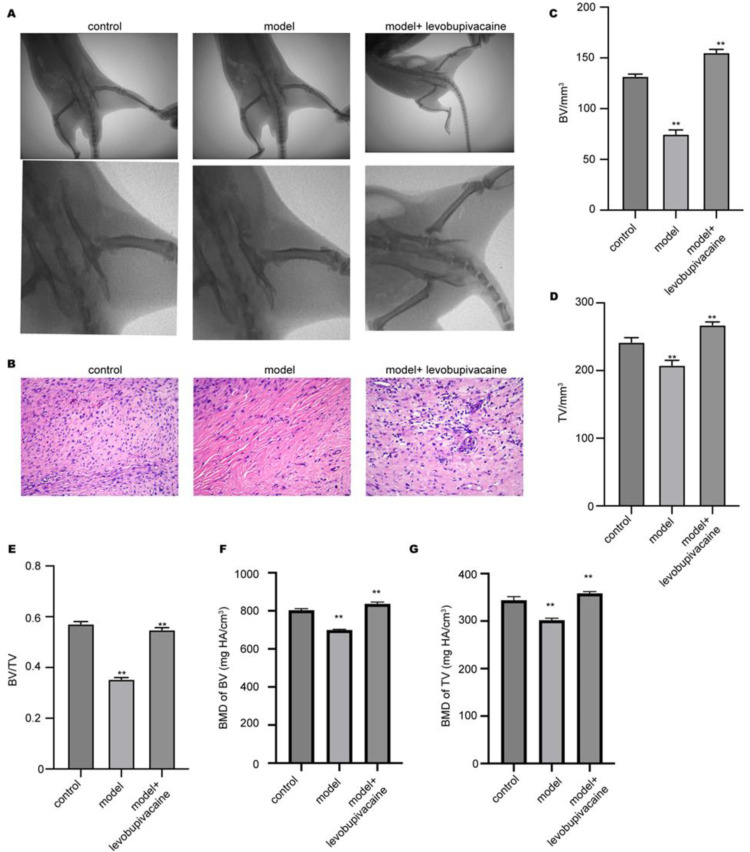
Levobupivacaine improves bone fracture healing. The rat bone fracture model was established (n = 6 rats in each group) and treated with levobupivacaine, the bone structure was analyzed at day 20. (A) Micro-CT imaging of the femur structure. (B) HE staining of bone tissues. (C‒G) Analysis of micro-CT parameters including BV, TV, BV/TV ratio, BMD/BV ratio, and BMD/TV ratio. **p *<* 0.01.

The authors first established a femur fracture model to determine the levobupivacaine effect on bone fracture healing. The results from micro-CT showed a larger callus structure at the injury site at day 20 in the group with treated compared with the model group (Fig. 1A). The histological analyses of the fracture site by HE-staining presented accelerated fracture healing under levobupivacaine administration compared with the model group (Fig. 1B), manifested by formed cartilage and external callus. The evaluation of micro-CT parameters revealed that the femur in the model group exhibited decreased Bone Volume (BV) (Fig. 1C), Total Volume (TV) (Fig. 1D), BV/TV ratio (Fig. 1E), Bone Mineral Density (BMD)/BV ratio (Fig. 1F), and BMD/TV ratio (Fig. 1G), whereas treatment with levobupivacaine elevated these parameters, suggesting recovered bone structure and function.

### Levobupivacaine promotes migration and proliferation of BMSCs

**Fig. 2:**
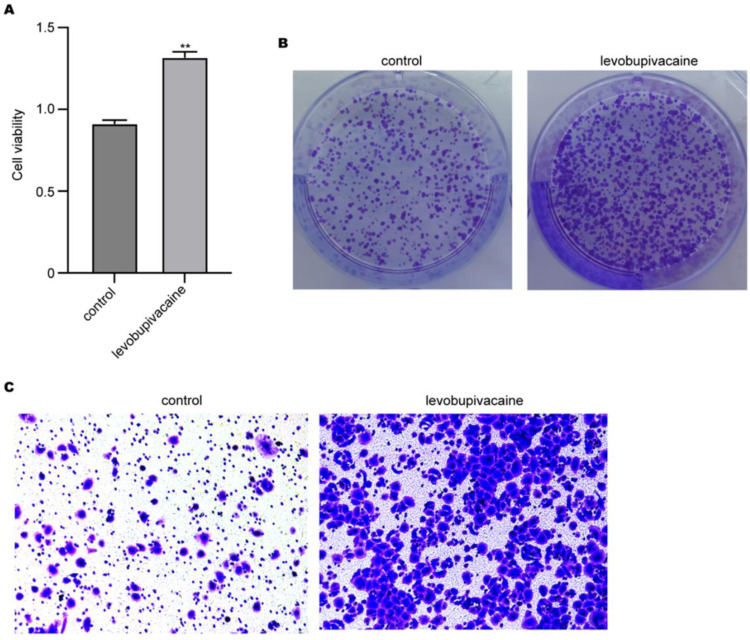
Levobupivacaine promotes migration and proliferation of BMSCs. BMSCs were treated with levobupivacaine for 24 h. Cell viability, proliferation, and migration were measured by (A) CCK-8, (B) Colony formation, and (C) Transwell experiment (** p *<* 0.01).

Next, the authors evaluated the effects of levobupivacaine on BMSCs. The results from colony formation and CCK-8 showed that levobupivacaine increased the relative viability of BMSCs (Fig. 2A) and the colony number formed by BMSCs (Fig. 2B). Besides, treatment with levobupivacaine notably elevated the number of BMSCs that migrated through Transwell chambers (Fig. 2C). These data indicated that levobupivacaine enhanced the migration and proliferation of BMSCs.

### Levobupivacaine stimulates HIF-1α/Ca^2+^signaling

**Fig. 3:**
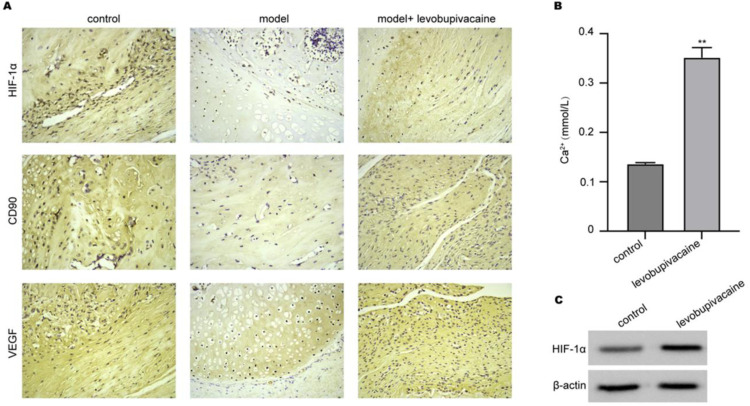
Levobupivacaine stimulates HIF/Ca^2+^signaling. (A) Expression of VEGF, HIF-1α, and CD90 in bone tissues was measured by IHC analysis. (B) Calcium concentration in blood samples collected from rats was measured. (C) The HIF-1α expression in BMSCs was measured by western blotting assay (** *p<* 0.01).

To identify the HIF/Ca^2+^ signaling role in fracture healing, the authors examined the CD90, HIF-1α, and VEGF expression in bone tissues collected from rats using an IHC experiment. As shown in Fig. 3A, the expression of VEGF, HIF-1α, and stem cell surface biomarker CD90 protein in the bone fracture model was markedly decreased in the model group, which was recovered by levobupivacaine treatment. Besides, the concentration of Ca^2+^ in blood samples was significantly increased by levobupivacaine (Fig. 3B). Moreover, the authors monitored the HIF-1α expression in BMSCs and confirmed that levobupivacaine treatment could enhance HIF-1α expression in vitro (Fig. 3C).

### Levobupivacaine modulates BMSCs phenotypes via targeting HIF-1α/Ca^2+^signaling

**Fig. 4:**
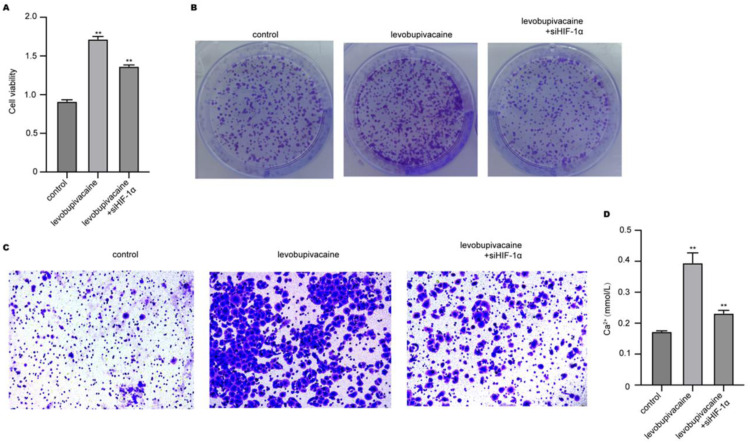
Levobupivacaine modulates BMSCs phenotypes via targeting HIF/Ca^2+^signaling. BMSCs were treated with levobupivacaine and transfected with siHIF-1α. Proliferation, cell viability, and migration were measured by (A) CCK-8, (B) colony formation, and (C) Transwell experiment. (D) The intracellular calcium concentration of BMSCs was measured (** *p<* 0.01).

To identify the HIF-1α role in BMSC migration and proliferation, the authors conducted an HIF-1α knockdown experiment. The experiment results from colony formation and CCK-8 indicated that knockdown of HIF-1α suppressed the levobupivacaine-induced BMSC viability and proliferation (Fig. 4A and B). The levobupivacaine-stimulated migration of BMSCs was also suppressed upon HIF-1α depletion (Fig. 4C). Moreover, knockdown of HIF-1α caused a significant decrease in levobupivacaine-elevated Ca^2+^ level (Fig. 4D).

## Discussion

Bone fracture healing encompasses processes such as cartilage formation and bone remodeling, which are closely correlated with the differentiation and function of MSCs.^23^ Besides, the marrow stromal elements, resident bone cells, and vascular structures are involved in the healing of bone trauma.^24^ BMSCs can differentiate into various cell types, including chondrocytes, vascular endothelial cells, osteoblasts, and, upon specific stimulation. In this work, the authors reported that levobupivacaine, the local anesthetic, exhibited promoting role in bone fracture healing via stimulating the proliferation and migration of BMSCs.

Levobupivacaine has a dose-dependent duration of anesthesia and is long-acting. With different aesthetic procedures, the start of effect is within 15-minutes. Levobupivacaine produced a sensory block for up to 9 h following an epidural dose of ≤202.5 mg, 6.5 h following intrathecal administration of 15 mg, and 17 h following brachial plexus block with 2 mg/kg in studies involving surgical anesthesia in adults. When paired with clonidine, morphine, or fentanyl, levobupivacaine proved to be particularly beneficial in managing postoperative pain. In addition, Levobupivacaine has recently been reported to directly participate in the treatment of diseases, such as cancers, via regulating cell proliferation, metastasis, and metabolism, and so on.^25^ For instance, therapy with levobupivacaine inhibited the development and spread of melanoma cells.^26^ In prostate cancer cells, levobupivacaine treatment suppressed glycolysis and oxidative phosphorylation of cells.^27^ Wang and colleagues recently reported that Levobupivacaine epigenetically suppressed gene expression by downregulating KAT5, which enhanced cell apoptosis and reduced osteosarcoma cell invasion and migration.^28^ Furthermore, levobupivacaine exhibited a positive effect on wound healing at the early period.^29^ Consistent with the therapeutic effects of levobupivacaine, the authors found that levobupivacaine administration could notably increase the bone volume and mineral density at day 20, indicating improved fracture healing.

Histological analysis further revealed that this process is accompanied by elevated VEGF, HIF-1α, and CD90 expression. The high levels of HIF-1α and VEGF indicated enhanced angiogenesis.^30^ Furthermore, it is widely known that VEGF promotes angiogenesis, a critical stage in the repair of bone fractures.^26^ In addition, HIF-1α is imperative for the migration of progenitor cells.^31^^,^^32^ Following levobupivacaine treatment, there was an increased expression of CD90 protein, a surface marker of stem cells, at the site of a bone fracture. Additionally, this high level of CD90 indicated a greater quantity of BMSCs.^33^^,^^34^ Previous studies have demonstrated that HIF-1α transcriptionally regulates the expression of key calcium channels such as Transient Receptor Potential (TRP) channels and calcium pumps, as well as modulates proteins associated with Store-Operated Calcium Entry (SOCE). These mechanisms collectively contribute to the regulation of intracellular Ca^2+^ homeostasis, suggesting that HIF-1α activation elevates cytosolic Ca^2+^ levels.^35^^,^^36^ In this study, when the HIF-1α gene was depleted by siRNAs, the level of HIF-1α and concentration of intracellular Ca^2+^ were suppressed, accompanied by suppressed BMSCs proliferation and migration. These findings indicated that HIF-1α-induced Ca^2+^ is regulated by levobupivacaine and is mandatory for BMSCs induction.

A limitation of this in vivo study is the absence of a positive control group treated with a known osteogenic agent such as BMP-2. While the present results demonstrate a significant pro-healing effect of levobupivacaine compared to an untreated fracture, future studies directly comparing its efficacy to standard treatments will be crucial to fully contextualize its therapeutic potential.

## Conclusion

The application of local anesthetics, like levobupivacaine, to promote the healing of hip fractures is one area of study that is relatively recent. Local anesthetics have traditionally been used primarily to reduce pain during orthopedic procedures. However, the current study suggests that levobupivacaine may have benefits beyond pain management, potentially influencing bone repair and recovery. An interesting theory that combines the domains of pharmacology, bone biology, and cellular signaling is that levobupivacaine can accelerate the healing of hip fractures by focusing on the Ca^2+^ signaling pathway. The present study suggested that levobupivacaine facilitates bone healing by promoting the proliferation and migration of BMSCs in a rat model, and regulates BMSCs phenotypes through activating the HIF-1α/Ca^2+^ signaling. These findings propose the clinical potential of levobupivacaine in the treatment of bone fractures.

## Funding

This research was funded by Key Research and Development 10.13039/501100014988Program of Shaanxi Province (2019SF-049).

## Data availability

The datasets generated and/or analyzed during the current study are available from the corresponding author upon reasonable request.

## CRediT authorship contribution statement

**Hui Zhang:** Writing – original draft. **Ying Zhang:** Data curation, Formal analysis. **Fang Wang:** Data curation, Formal analysis. **Yulong Wei:** Conceptualization, Writing – original draft, Funding acquisition. **Dezhi Wang:** Conceptualization, Writing – original draft, Funding acquisition.

## Declaration of competing interest

The authors declare no conflicts of interest.
